# P57 Prevalence of antimicrobial resistance in acute community-acquired urinary tract infections in Sub-Saharan Africa: a systematic review

**DOI:** 10.1093/jacamr/dlaf230.064

**Published:** 2025-12-04

**Authors:** Amelia Williams-Walker, Lucy Lammie, Catrin E Moore

**Affiliations:** City St George’s University, London, UK; City St George’s University, London, UK; City St George’s University, London, UK

## Abstract

**Background:**

Urinary tract infections (UTIs) are one of the most common bacterial infections globally and a leading cause for outpatient visits. In Sub-Saharan Africa, widespread over the counter dispensing of antibiotics and limited healthcare resources contribute to high levels of antimicrobial resistance (AMR) in the community. However, data on AMR in community-acquired UTIs remain scarce. This systematic review addresses this knowledge gap by synthesizing the available evidence on AMR in this patient population.

**Objectives:**

We aimed to determine the resistance rates of the most common pathogens causing community-acquired UTIs in Sub-Saharan Africa to the antibiotics most frequently prescribed for their treatment. Additionally, we sought to evaluate whether resistance patterns varied geographically or temporally within the region.

**Methods:**

A systematic search was conducted for peer-reviewed literature published between 1 January 2000 and 1 November 2024 using Medline, Embase and Global Health databases. The search strategy captured studies evaluating AMR in community-acquired UTIs within the target region. Exclusion criteria included: studies with less than 10 bacterial isolates and those involving patients with comorbidities that predispose them to complicated infections. Studies were screened independently by two reviewers at the title, abstract and full text stages before extracting data onto a standardized spreadsheet. Quality assessment was done using an adapted descriptive tool that considered laboratory methodology and data completeness. Random-effects meta-analysis and forest plots analysed resistance rates of *Escherichia coli* and *Klebsiella* spp., the most common pathogens, against five antibiotics most frequently used for treatment.

**Results:**

The systematic database search identified 2271 articles for screening; 41 studies from 19 countries across Sub-Saharan Africa met the inclusion criteria. These studies contained antimicrobial susceptibility data on 6245 *E. coli* and 930 *Klebsiella* spp. isolates with 10 antibiotic-organism pairs (2 bacterial species and 5 antibiotics) selected for analysis. Among the antibiotics examined, trimethoprim–sulfamethoxazole demonstrated the highest resistance rates with 63.2% (Standard Error [SE]=5.4) of *E. coli* and 66.4% (SE=10.1) of *Klebsiella* spp. Nitrofurantoin showed significantly lower resistance rates: 8.2% (SE=4.6) and 28.3% (SE=8.2), respectively. *E. coli* resistance to ceftriaxone (30.8%, SE=5.8) and ciprofloxacin (32.8%, SE=3.9) was also significantly lower than trimethoprim/sulfamethoxazole resistance. Heterogeneity was consistently very high (>95%) across all antibiotic-organism pairs, seemingly driven by different UTI definitions and laboratory methodologies. Subgroup analyses revealed that geography (country or region) did not account for this variability. When stratified by time (5 year groups), only *E. coli* resistance to amoxicillin/clavulanic acid showed a significant reduction in heterogeneity (Q test for moderators, *P*=0.013).

**Conclusions:**

In Sub-Saharan Africa, data on AMR in community-acquired UTIs are severely limited and most countries lack eligible studies. Included studies utilized a variety of laboratory definitions for UTI positivity and antimicrobial susceptibility testing guidelines. Nitrofurantoin has the lowest observed resistance rates, in line with the African Standard Treatment Guidelines for UTI management. Given high trimethoprim/sulfamethoxazole resistance rates, its empirical use should be reconsidered. We found no temporal trends in resistance patterns over the 24 years or a significant geographical variation within Sub-Saharan Africa.

